# 6-gingerol promotes apoptosis of ovarian cancer cells through miR-506/Gli3 signaling pathway activation

**DOI:** 10.3389/fonc.2025.1547771

**Published:** 2025-09-12

**Authors:** Jun Xiong, Hong-Hu Wu, Hui Jiang, Huan Li, Xiao-Qing Tan, Xiao-Ju He, Xue-Xin Cheng

**Affiliations:** ^1^ Department of Obstetrics and Gynecology, The Second Affiliated Hospital of Nanchang University, Nanchang, Jiangxi, China; ^2^ Biological Resource Center, The Second Affiliated Hospital of Nanchang University, Nanchang, Jiangxi, China; ^3^ Nanchang University, Nanchang, China; ^4^ Jiangxi Provincial Key Laboratory of Preventive Medicine, School of Public Health, Nanchang University, Nanchang, China

**Keywords:** ovarian cancer, 6-gingerol, apoptosis, miR-506, Gli3

## Abstract

**Purpose:**

Ginger rhizomes have shown potential for promoting human health, including the prevention and treatment of cancer. Here, we investigated the anticancer activities of 6-gingerol and explored its mechanisms of action in ovarian cancer cells.

**Methods:**

SKOV3 ovarian cancer cells were treated with different concentrations of 6-gingerol. Clonogenic assays, Flow cytometry, and Western blotting were used to evaluate cell survival and apoptosis. RT-qPCR and transfection experiments were performed to assess the role of miR-506, and bioinformatics tools were used to identify Gli3 as a target gene.

**Results:**

*In vitro*, ovarian cancer cells underwent apoptosis following 6-gingerol treatment. 6-Gingerol suppressed Gli3 expression without affecting Bax, Bcl-2, or Bcl-xL levels. Low miR-506 expression was observed in ovarian cancer tissues, whereas 6-gingerol significantly promoted its expression. miR-506 directly suppressed Gli3 expression and induced apoptosis in SKOV3 cells.

**Conclusions:**

Our results indicate that gingerol promoted the upregulation of miR-506, leading to the induction of apoptosis in ovarian cancer cells. This study supports the potential of 6-gingerol-based therapy for ovarian malignancies.

## Introduction

Ovarian cancer is the seventh most prevalent cancer in women and has the highest mortality rate among gynecological cancers (1). The five-year survival rate in patients with ovarian cancer is approximately 47% (2, 3). Due to the lack of specific and sensitive early detection methods, ovarian cancer is often diagnosed at an advanced stage when metastasis has already occurred, limiting the effectiveness of surgical treatments and chemotherapy (4–7). Although poly (ADP-ribose) polymerase inhibitors show promise, further clinical and laboratory studies are required to confirm their therapeutic efficacy (8, 9). Therefore, identifying new therapeutic targets for ovarian cancer is crucial.

Natural compounds with anticancer properties have shown effectiveness against various cancer types, often with minimal side effects (10, 11). Ginger (*Zingiber officinale* Roscoe) is a rich source of bioactive phytochemicals, with 6-gingerol being the primary phenolic compound. 6-Gingerol exhibits anti-inflammatory, anti-proliferative, and antioxidant effects (12–14). It stimulates antitumor activity in breast and cervical cancer, among other cancer types (15). However, the effects and mechanisms of 6-gingerol on ovarian cancer cell growth remain largely unknown.

This study aimed to determine whether 6-gingerol exerts anticancer effects on human ovarian cancer cells. We focused on the molecular mechanisms via which 6-gingerol suppresses cell growth and progression through the induction of apoptosis. Our findings revealed a strong correlation between Gli3 downregulation and 6-gingerol-induced apoptosis. Additionally, we confirmed that miR-506 is expressed at low levels in ovarian cancer tissues. By inhibiting Gli3 expression, miR-506 promotes apoptosis in human ovarian cancer cells. Furthermore, treatment with an miR-506-specific inhibitor reversed the cytotoxic effects of 6-gingerol. In conclusion, we investigated the effects of 6-gingerol on ovarian cancer cell proliferation and explored the underlying molecular mechanisms. Our study identified the miR-506/Gli3 signaling axis as a key pathway through which 6-gingerol induces apoptosis in ovarian cancer cells.

## Methods and materials

### Cell culture

The SKOV3 human ovarian carcinoma cell line was obtained and authenticated by the American Type Culture Collection (Manassas, VA, USA). The cells were cultured in Dulbecco’s modified Eagle medium (Invitrogen, USA) supplemented with 10% fetal bovine serum (Invitrogen), 1% streptomycin, and 1% ampicillin. Cells were maintained at 37°C in a humidified incubator with 5% CO_2_. 6-Gingerol was purchased from Sigma-Aldrich (G1046).

### Cell transfection

Transfection was performed using Lipofectamine 3000 (Invitrogen) following the manufacturer’s protocol. Specifically, 2 µg of plasmids were transfected into cells that had been seeded on a six-well plate in the log phase 24 h prior. The transfection was performed using Lipofectamine 2000, and GFP transfection was used in parallel to estimate transfection efficiency. The pcDNA3.1-miR-506 plasmid and its scrambled negative control were obtained from GenePharma (Shanghai, China).

### Clonogenic survival assay

Cells (1000 per dish) were seeded in triplicate in 100 mm Petri dishes and cultured in RPMI-1640 medium for 9 consecutive days. The medium was completely replaced on the day of seeding. Cells were fixed in 100% cold methanol for 15 min and stained with 0.25% crystal violet for another 15 min at room temperature. Colonies were washed with PBS and counted in three random fields.

### PCR analysis

Total RNA was extracted using a HiPure Universal miRNA kit (Magen, Guangzhou, China) according to the manufacturer’s instructions. RNA quality and quantity were verified using a BioAnalyzer 2100 (Agilent, Santa Clara, CA, USA). cDNA was synthesized using a miScript Reverse Transcription Kit (Qiagen, Valencia, CA, USA). Real-time PCR was performed using a CFX Connect™ Real-Time System (Bio-Rad, Inc., Hercules, CA, USA) and a miScript PCR Kit (Qiagen) according to the manufacturers’ instructions. Relative miR-506 expression was normalized to that of U6 rRNA and calculated using the 2^-ΔΔCt^ method. Moreover, 5s rRNA was used for normalization to determine relative expression. Primers were synthesized by GenePharma (Shanghai, China). The following qPCR primers were used: miR-506 forward: 5′-GATCCTCTACTCAGAAGGGTGCCTTATTTTTG-3′; miR-506 reverse: 5′-AATTCAAAAATAAGGCACCCTTCTGAGTAGAG-3′; U6 forward: 5′-CTCGCTTCGGCAGCACA-3′; and U6 reverse: 5′-CGAATTTGCGTGTCATCCT-3′.

### Western blotting

Total protein was extracted using a radioimmunoprecipitation assay, and concentrations were determined using a Pierce BCA Protein Assay kit (Thermo Fisher Scientific, Inc.), according to the manufacturer’s instructions. Proteins (30 µg/lane) were separated using 10% sodium dodecyl sulfate-polyacrylamide gel electrophoresis (SDS-PAGE) and transferred to polyvinylidene difluoride membranes (EMD Millipore, Billerica, MA, USA). Membranes were blocked with 5% non-fat milk in PBS with 0.05% Tween-20 (PBST) and incubated overnight with primary antibodies at 4°C. Detection was performed using enhanced chemiluminescence (ECL, Millipore) after incubation with the secondary antibodies and a wash with Tris-buffered saline. The antibodies used were anti-rabbit (ab6721, 1:2500) and anti-mouse (ab6789, 1:2500) (both from Abcam).

### Cell apoptosis analysis

Apoptosis was analyzed using annexin V/propidium iodide (PI) staining and flow cytometry (BD Biosciences, Franklin Lakes, NJ, USA). Cells in a single-cell suspension were incubated in the dark for 15 min in HEPES buffer and analyzed using ModFit software (BD Biosciences).

### Caspase inhibition assay

To determine whether apoptosis induced by 6-gingerol is caspase-dependent, SKOV3 cells were pre-treated with 20 µM Z-VAD-FMK (Selleck Chemicals) for 2 hours, followed by treatment with 20 µM 6-gingerol. Apoptosis was then assessed using Annexin V-FITC/PI staining.

### Statistical analysis

Unless otherwise stated, all experiments were performed at least three times independently. Data are presented as mean ± standard deviation (SD). Statistical analyses were performed using SPSS 11.5 (SPSS Inc., Chicago, IL, USA). One-way ANOVA and multiple t-tests were used to assess significance, with *P* < 0.05 considered statistically significant.

## Results

### 6-gingerol induced apoptosis in SKOV3 cells

**Figure 1:**
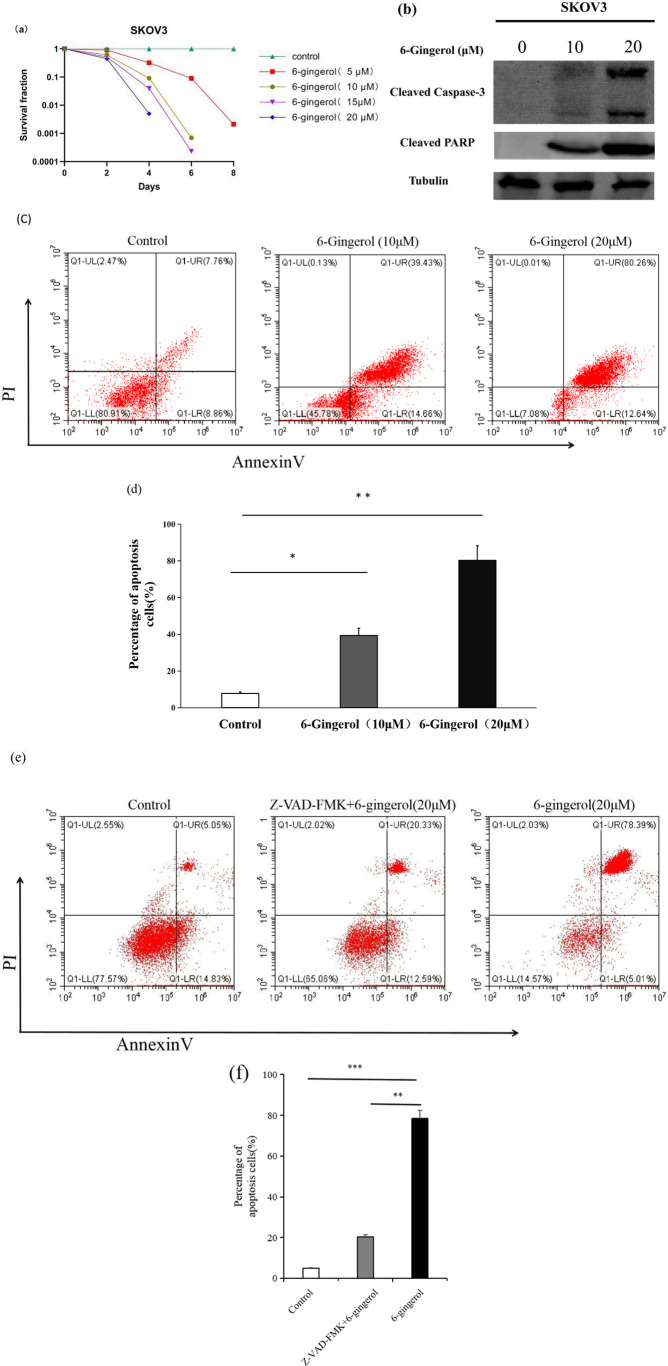
6-gingerol induces apoptosis in SKOV3 cells. **(a)** Clonogenic survival assay showing the survival rates of SKOV3 cells treated with 5 µM,10 µM,15 µM and 20 µM 6-gingerol for different durations (1^st^, 2^nd^, 4^th^, and 6^th^ days). Results are based on independent experiments (n = 3). **(b)** Western blot analysis of cleaved caspase-3 or and cleaved PARP levels in SKOV3 cells treated with 6-gingerol. Tubulin was used as the loading control. **(c)** Flow cytometry analysis of apoptosis in SKOV3 cells treated with different 6-gingerol concentrations, using an Annexin V-FITC & propidium iodide (PI) apoptosis kit. Results are from three independent experiments (n = 3). **(d)** Quantification of apoptotic cells (double-positive for PI and Annexin V) from panel **(c)**. Results are presented as mean ± SD (n = 3). *P < 0.05, **P < 0.001. **(e)** Flow cytometry analysis of apoptosis in SKOV3 cells treated with 20 µM Z-VAD-FMK (Selleck Chemicals) for 2 hours, followed by treatment with 20 µM 6-gingerol and treated with 20 µM 6-gingerol directly for 2 days, using an Annexin V-FITC & propidium iodide (PI) apoptosis kit. Results are from three independent experiments (n = 3). **(f)** Quantification of apoptotic cells (double-positive for PI and Annexin V) from panel **P < 0.01, ***P < 0.001 **(e)**. Results are presented as mean ± SD (n = 3). *P < 0.05, **P < 0.001.

We conducted an *in vitro* evaluation to determine the potential cytotoxic effects of 6-gingerol on human ovarian carcinoma SKOV3 cells. SKOV3 cells were treated with 5 µM,10 µM,15 µM and 20 µM concentrations of 6-gingerol for 6 days, and their survival rates were assessed using a clonogenic assay. **Figure 1a** shows a significant decrease in clonogenic survivors at both concentrations. In the 5 µM group, the survival rates were91%, 3.2%, 0.9% and0.07% on the 2^nd^, 4^th^,6^th^ and8^th^ days of culture, respectively. In the 10 µM group, the survival rates were 61%, 9.1%, and 0.07% on the 2^nd^, 4^th^, and 6^th^ days of culture, respectively. In the 15 µM group, the survival rates were 52%, 0.39%, and 0.023% on the 2^nd^, 4^th^, and 6^th^ days of culture, respectively. In the 20 µM group, all cells died by the 6^th^ day of culture. To further confirm apoptosis, we analyzed the levels of cleaved caspase-3 and cleaved poly (ADP-ribose) polymerase (PARP) in response to 6-gingerol treatment, using endogenous tubulin as a loading control. As shown in **Figure 1b**, caspase-3 and cleaved PARP levels increased with higher 6-gingerol concentrations. To assess the dose-dependent effects of 6-gingerol on ovarian cancer cell apoptosis, we treated SKOV3 cells with 0, 10, and 20 µM of 6-gingerol for 2 days and analyzed the results using flow cytometry. The data (**Figures 1c, d**) show that the extent of apoptosis in SKOV3 cells increased proportionally with the 6-gingerol concentration. To further confirm the caspase dependence of 6-gingerol-induced apoptosis,SKOV3 cells were pre-treated with 20 µM Z-VAD-FMK (Selleck Chemicals) for 2 hours, followed by treatment with 20 µM 6-gingerol and treated with 20 µM 6-gingerol directly for 2 days. The data (**Figures 1e, f**) show that the extent of apoptosis in SKOV3 cells decreased proportionally with the Z-VAD-FMK treatment. These findings provide valuable insight into the caspase dependence of 6-gingerol to induce significant apoptotic responses in ovarian cancer cells, suggesting its effectiveness as a therapeutic agent.

### 6-gingerol reduces Gli3 expression

**Figure 2:**
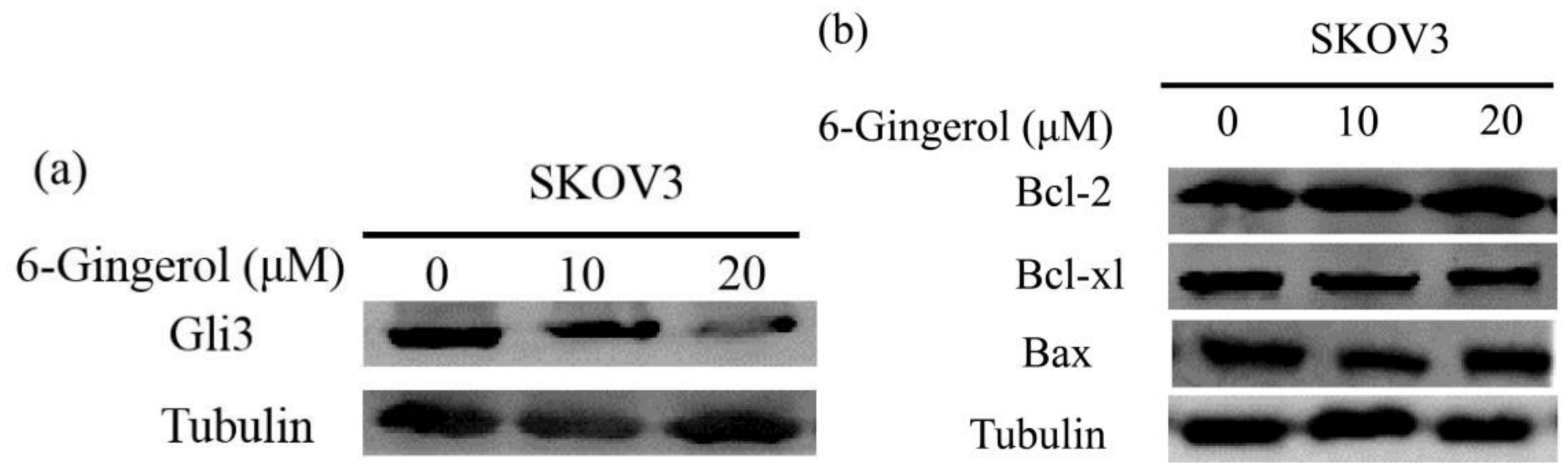
6-gingerol inhibits SKOV3 cells by reducing Gli3 expression. **(a)** Western blot analysis showing Gli3 protein levels in SKOV3 cells treated with 6-gingerol. Tubulin was used as a loading control. **(b)** Western blot analysis of apoptosis-related proteins (Bcl-xL, anti-Bcl-2, and Bax) in SKOV3 cells treated with 6-gingerol. Tubulin was used as a loading control.

Given that GL13 knockdown inhibits the growth and migration of ovarian cancer cells (16), we investigated Gli3 expression in 6-gingerol-induced apoptosis. As shown in **Figures 2a, b**, treatment with 6-gingerol significantly reduced Gli3 expression in SKOV3 cells. However, no notable changes in the levels of other apoptosis-related proteins, such as Bcl-2, Bcl-w, and Bik, were observed. These results suggest that Gli3 downregulation plays a critical role in 6-gingerol-induced apoptosis in ovarian cancer cells.

### 6-gingerol upregulates miR-506

**Figure 3:**
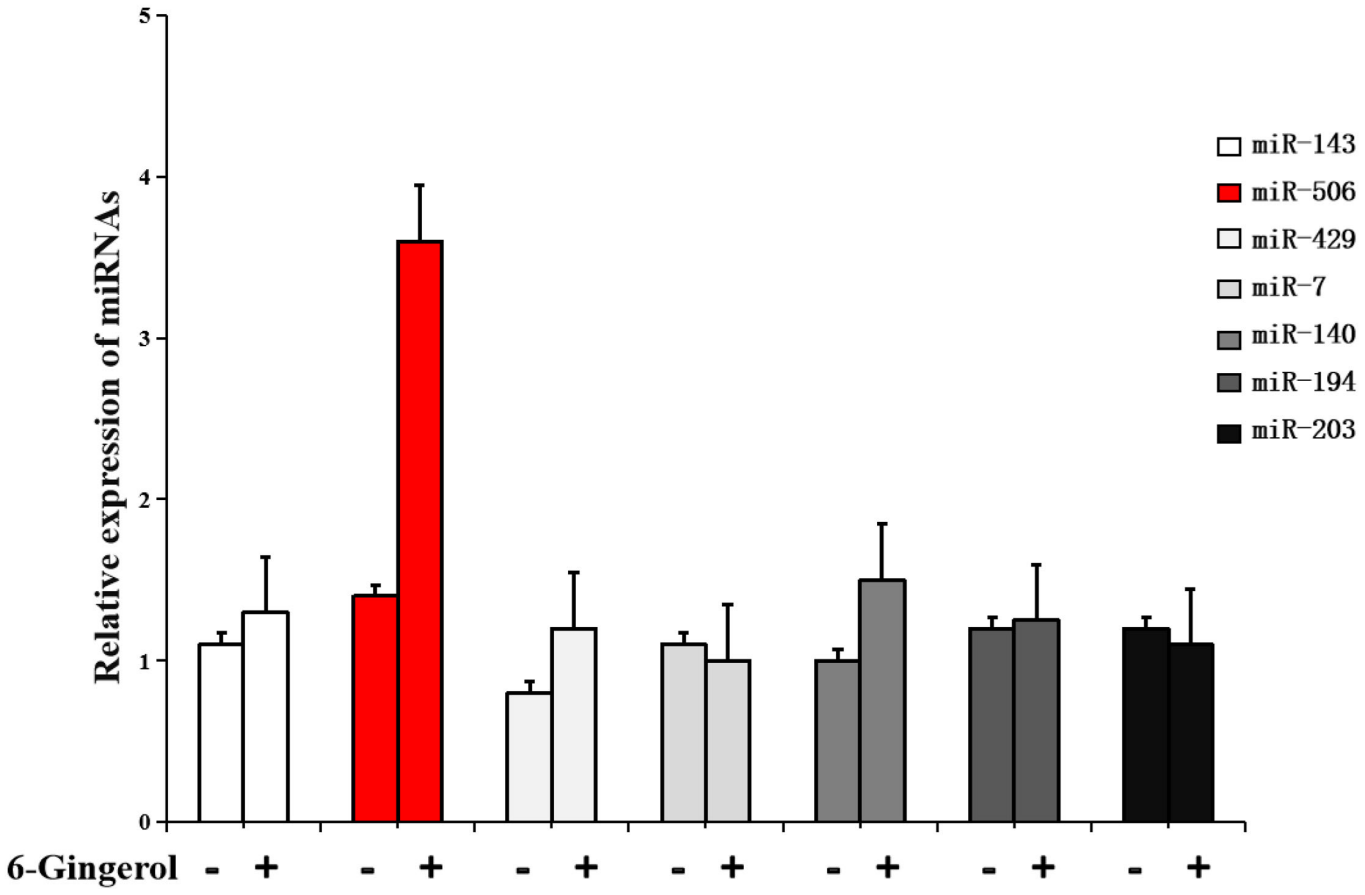
6-gingerol increases microRNA (miR)-506 expression in SKOV3 cells. RT-PCR analysis showing the expression levels of candidate microRNAs predicted to target Gli3 in SKOV3 cells treated with 6-gingerol. Data are normalized to the levels of 5s rRNA.

Evidence suggests that miRNAs are key regulators involved in cancer cell proliferation, differentiation, metastasis, and apoptosis. Therefore, we hypothesized that miRNAs might mediate the regulation of Gli3 expression by 6-gingerol. Using bioinformatics algorithms, including TargetScan, miRWalk, and miRDB, we identified seven candidate miRNAs that could potentially regulate Gli3 expression in response to 6-gingerol treatment. The relative expression of these miRNAs was determined using PCR and normalized to that of endogenous 5s rRNA. As shown in **Figure 3**, 6-gingerol treatment significantly upregulated miR-506 expression compared to other candidate miRNAs [(3.5 ± 0.6)-fold].

### miR-506 directly inhibits Gli3 and induces apoptosis in SKOV3 cells

**Figure 4:**
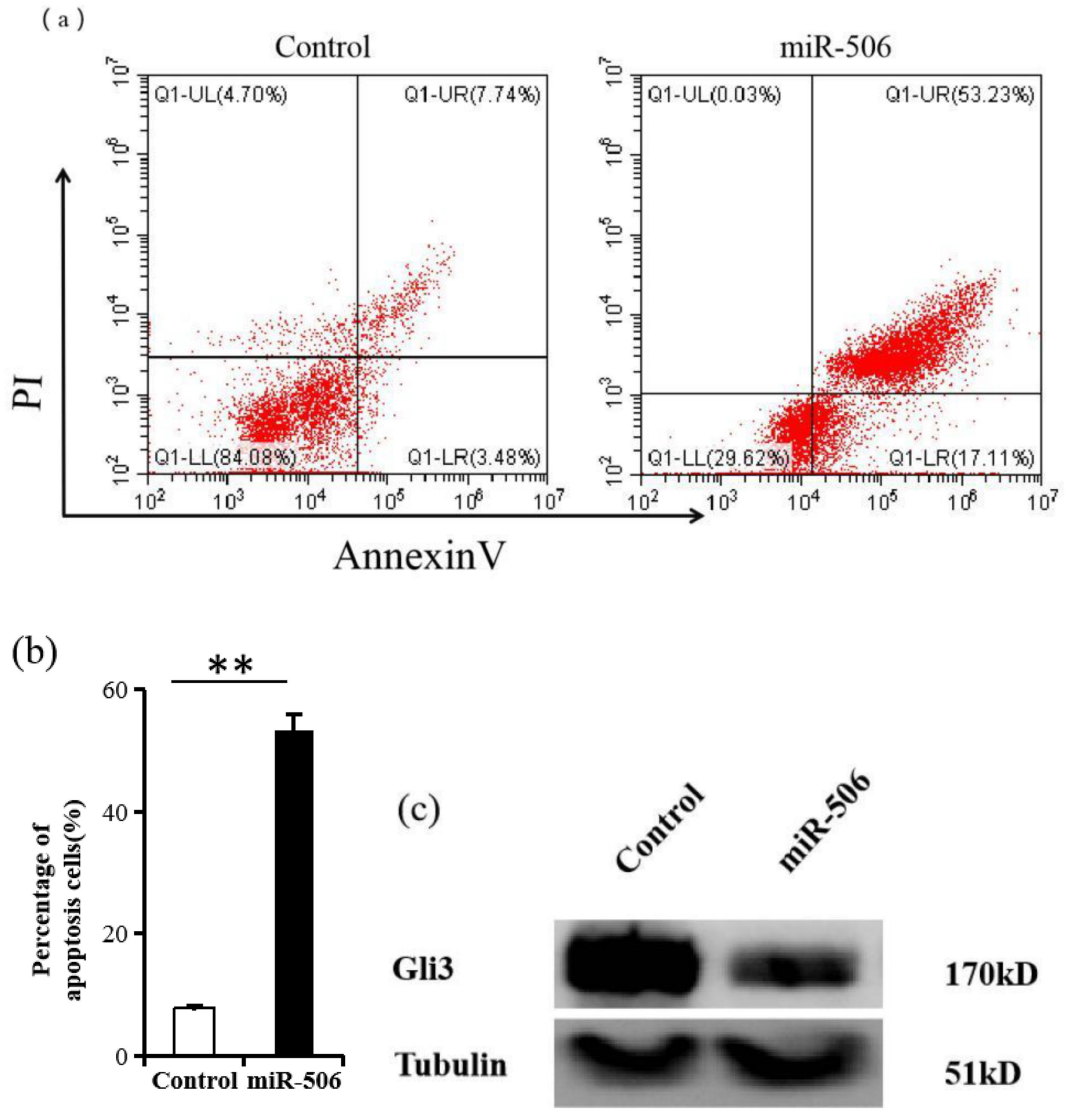
miR-506 suppresses Gli3 and induces apoptosis in SKOV3 cells. **(a)** Flow cytometry analysis of apoptosis in SKOV3 cells after transfection with miR-506, using Annexin V-FITC and propidium iodide (PI) staining. Results are based on three independent experiments (n = 3). **(b)** Quantification of apoptotic cells from panel **(a)**. The data show the percentage of double-positive Annexin V and PI cells. Results are presented as mean ± SD (n = 3). **P < 0.01. **(c)** Western blot analysis showing Gli3 protein levels. Tubulin was used as a loading control.

To verify the effect of miR-506 on Gli3 expression and apoptosis, we transfected SKOV3 cells with miR-506. As shown in **Figure 4a**, upregulation of miR-506 significantly increased apoptosis in SKOV3 cells (45.2% ± 5.1%) compared to that in the scramble control (3.7% ± 0.3%, **Figure 4b**). Western blot analysis further showed that excessive miR-506 levels suppressed Gli3 protein expression (**Figure 4c**).

### 6-gingerol induces apoptosis in SKOV3 cells via miR-506

**Figure 5:**
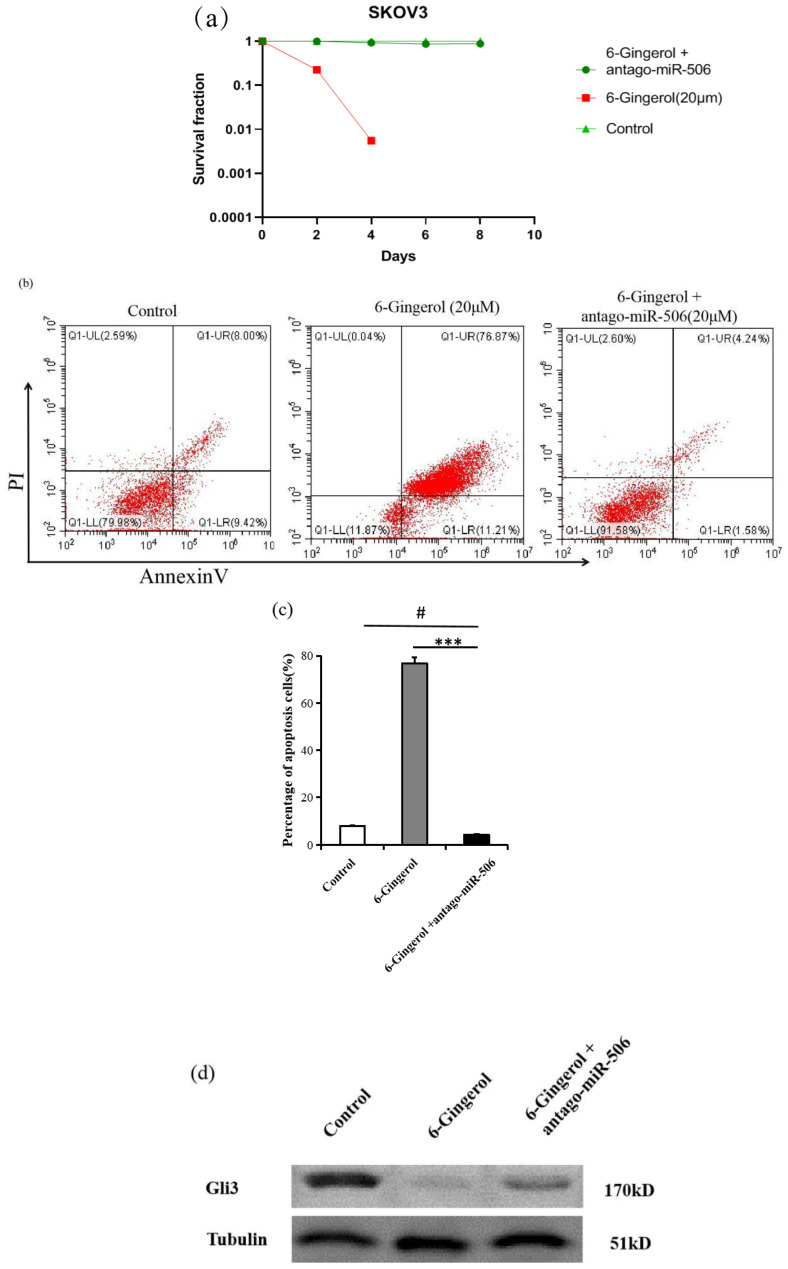
6-gingerol induces apoptosis in SKOV3 cells via miR-506. **(a)** Clonogenic survival assay showing the percentage of SKOV3 cells surviving after treatment with 20 μM 6-gingerol or 20 μM 6-gingerol + antago-miR-506 over different time points (days 1, 2, 4, 6, and 8). Results are based on three independent experiments (n = 3). **(b)** Flow cytometry analysis of apoptosis in SKOV3 cells treated with 6-gingerol or 6-gingerol + antago-miR-506 using Annexin V-FITC and propidium iodide (PI) staining (n = 3) # P > 0.05, *** P < 0.001. **(c)** Quantification of apoptotic cells (double-positive for PI and Annexin V) from panel **(b)**. Results are presented as mean ± SD (n = 3). ***P < 0.001, #P > 0.05. **(d)** Western blot was performed with anti-Gli3 antibody. Tubulin was used as a loading control.

We found that both 6-gingerol and miR-506 induced apoptosis in ovarian cancer cells. To investigate whether miR-506 mediates the apoptosis effects of 6-gingerol, we used an miR-506-specific antagonist (antago-miR-506). As shown in **Figure 5a**, treatment with 20 μM 6-gingerol significantly reduced the survival rate of SKOV3 cells. This effect was reversed by co-treatment with antago-miR-506. Similarly, flow cytometry analysis showed that the apoptosis induced by 6-gingerol in SKOV3 cells (68.2% ± 3.1%) was significantly reduced (9.4% ± 0.9%) when antago-miR-506 was introduced (*P*<0.05, **Figures 5b, c**). To elucidate the molecular mechanism, we performed western blot analysis to assess Gli3 expression in three groups: control, 6-gingerol, and 6-gingerol + antago-miR-506. As shown in **Figure 5d**, 6-gingerol treatment suppressed Gli3 expression; however, this suppression was reversed by antago-miR-506. These findings suggest that 6-gingerol induces apoptosis in SKOV3 cells by upregulating miR-506, which downregulates Gli3.

## Discussion

Conventional anticancer therapies often lack specificity, targeting not only cancer cells but also healthy cells, leading to severe side effects. For example, platinum-based chemotherapy for ovarian cancer frequently causes gastrointestinal distress, bone marrow suppression, and liver and kidney damage (17, 18). Targeted therapies, while more specific, can still produce adverse effects, such as hypertension, proteinuria, and reduced blood cell counts. Natural compounds have emerged as promising alternatives to traditional treatments, offering increased efficiency with fewer side effects. These compounds can specifically target oncogenes and may also synergize with other chemotherapeutic agents (19, 20).

Throughout history, plant-based remedies have been widely used to treat various diseases, a practice that remains relevant today. Currently, herbal drugs account for over 50% of therapies in clinical trials (21). 6-Gingerol, the most abundant and biologically active phenolic compound present in the roots of ginger (*Zingiber officinale*), which has been more studied and more bioavailable than other phenolic compounds in ginger, exemplifies the medicinal potential of such natural products. Ginger has been used for centuries in China as a culinary spice and medicinal remedy. Ginger has been a staple in traditional Chinese medicine for centuries, valued for its anti-inflammatory, antibacterial, and anticancer properties. Notably, 6-gingerol induces apoptosis in breast cancer cells by activating Bax transcription and caspase-7 (22).

The ability of 6-gingerol to arrest the cell cycle and induce apoptosis has been shown in human cervical and oral cancer cells (23, 24). Furthermore, 6-gingerol exhibits cytoprotective effects by reducing apoptosis and oxidative stress, potentially via the activation of Nrf2 pathways and inhibition of p38/NF-κB signaling (25). However, the mechanisms underlying the cytotoxic effects of 6-gingerol in ovarian cancer cells were previously unclear. Our study demonstrates that a concentration of 10 μM 6-gingerol effectively suppresses the clonogenic capacity of SKOV3 cells, leading to apoptosis.

We identified Gli3, a zinc-finger transcription factor, as a key player in this process. Gli3 has been implicated in the growth and metastasis of several cancer types. Knockdown of Gli3 suppresses the proliferation and migration of androgen receptor-positive breast and ovarian cancer cells, which does not occur for androgen receptor-negative cells (16). Additionally, loss of Gli3 in fibroblasts reduces suppressor cells derived from myeloid lineages and enhances natural killer cell activity, thereby inhibiting tumor growth (26). In colorectal cancer, Gli3 knockdown reduces cell migration and invasion by affecting epithelial-mesenchymal transition through the ERK signaling pathway. Elevated Gli3 expression correlated with poor prognosis in patients with colorectal cancer (27, 28). These results complicate the role of Gli3 expression in tumor tissues. In our study, 6-gingerol treatment significantly reduced Gli3 protein levels in SKOV3 cells. Interestingly, the expression of other apoptosis-related proteins, such as Bcl-2, Bax, and Bcl-xL, remained unchanged. To further explore the regulation of Gli3, we examined the role of miR-506, a microRNA known to regulate cell growth, differentiation, and metastasis, in SKOV3 cells treated with 6-gingerol. Bioinformatics analysis predicted miR-506 as a potential regulator of Gli3 expression, and our results confirmed that 6-gingerol upregulates miR-506, which in turn suppresses Gli3 expression and induces ovarian cancer cell apoptosis.

The role of miR-506 in cancer is context-dependent. In some cancer types, miR-506 acts as a tumor suppressor, whereas in others, it may function as an oncogene (29). For instance, Tong et al. (30) reported a high miR-506 expression in HCPT-resistant SW1116/HCPT colon cancer cells, suggesting its role in tumor suppression. Similarly, Streicher et al. (31) showed that the miR-506–514 cluster is consistently overexpressed in most melanomas, independent of the presence of B-raf or N-ras mutations. This cluster, or one of its sub-clusters (Sub-cluster A) comprising six mature miRNAs, can inhibit cell growth, promote apoptosis, and reduce invasiveness and colony formation in melanoma cell lines by reducing the expression of its target genes. Conversely, Luo et al. (32) found that miR-506 expression is reduced in glioblastoma. Overexpression of miR-506 in these cells suppressed cell growth, blocked the G1/S cell cycle transition, and inhibited cell invasion into glioblastoma cells. Zhang et al. (33) reported that cancer tissues and cultured cells exhibited lower miR-506 levels. They found that miR-506 expression was negatively correlated with EZH2 expression, lymph node invasion, tumor growth, metastasis, and TNM stage. Higher miR-506 levels were associated with a more favorable prognosis in patients. Consistent with these findings, we observed that miR-506 expression was significantly downregulated in ovarian cancer tissues. Our results showed that upregulation of miR-506 reduces ovarian cancer cell proliferation by targeting the transcription factor Gli3.

This study has several limitations. First, although SKOV3 cells are representative of high-grade serous ovarian cancer, validation in additional cell lines (e.g., CAOV3, OVCAR3) would strengthen the findings. Second, the functional role of Gli3 in migration/invasion was not examined, which should be addressed in future studies given its known metastatic functions. These limitations do not affect the core mechanistic conclusions but highlight directions for further research.

In summary, Our findings demonstrate that 6-gingerol induces ovarian cancer cell apoptosis through miR-506-mediated Gli3 suppression, providing an alternative to conventional Bax/Bcl-2-targeting approaches. Interestingly, while 6-gingerol has shown promise in combination with cisplatin (34), our work reveals its equally potent single-agent activity through this newly identified pathway. The clinical relevance of miR-506 downregulation in patient tumors further supports the therapeutic potential of 6-gingerol, particularly for tumors with impaired miR-506/Gli3 regulation.

## Data Availability

The datasets presented in this study can be found in online repositories. The names of the repository/repositories and accession number(s) can be found in the article/supplementary material.
